# Supplementary material for: Conditional chemoconnectomics (cCCTomics) as a strategy for efficient and conditional targeting of chemical transmission
Source: eLife. 2024 Apr 30;12:RP91927. doi: 10.7554/eLife.91927 (PMC11060718; doi:10.7554/eLife.91927)
Supplement: Figure 3—source data 3. [file elife-91927-fig3-data3.zip › Figure 3 source data3 part 1.zip!/Figure 3-Source data 3-part1/1063-13-CRISPResso_Report_8cz_fsdr.zip!/CRISPResso_on_1063_13/1c.Alignment_barplot.pdf]

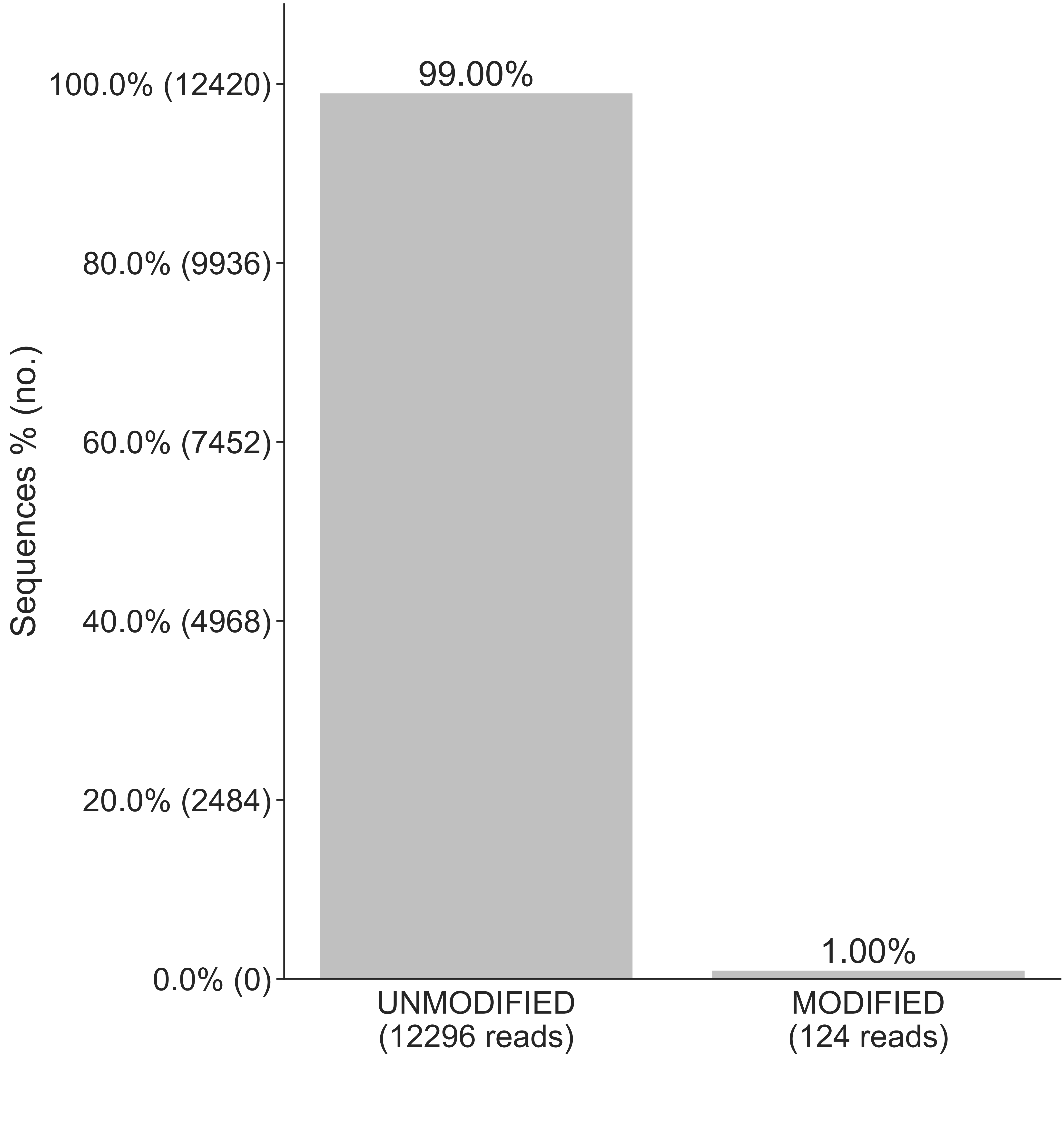

Sequences % (no.)
100.0% (12420)
80.0% (9936)
60.0% (7452)
40.0% (4968)
20.0% (2484)
0.0% (0)
99.00%
1.00%
UNMODIFIED
(12296 reads)
MODIFIED
(124 reads)
